# Atomic scale dynamics of a solid state chemical reaction directly determined by annular dark-field electron microscopy

**DOI:** 10.1038/srep07555

**Published:** 2014-12-22

**Authors:** Timothy J. Pennycook, Lewys Jones, Henrik Pettersson, João Coelho, Megan Canavan, Beatriz Mendoza-Sanchez, Valeria Nicolosi, Peter D. Nellist

**Affiliations:** 1 SuperSTEM Laboratory, STFC Daresbury, Keckwick Lane, Warrington WA4 4AD, United Kingdom; 2 Department of Materials, University of Oxford, Parks Road, Oxford OX1 3PH, United Kingdom; 3 Centre for Research on Adaptive Nanostructures and Nanodevices (CRANN), Trinity College Dublin, Dublin 2, Ireland; 4 School of Physics, Trinity College Dublin, Dublin 2, Ireland; 5 School of Chemistry, Trinity College Dublin, Dublin 2, Ireland

## Abstract

Dynamic processes, such as solid-state chemical reactions and phase changes, are ubiquitous in materials science, and developing a capability to observe the mechanisms of such processes on the atomic scale can offer new insights across a wide range of materials systems. Aberration correction in scanning transmission electron microscopy (STEM) has enabled atomic resolution imaging at significantly reduced beam energies and electron doses. It has also made possible the quantitative determination of the composition and occupancy of atomic columns using the atomic number (Z)-contrast annular dark-field (ADF) imaging available in STEM. Here we combine these benefits to record the motions and quantitative changes in the occupancy of individual atomic columns during a solid-state chemical reaction in manganese oxides. These oxides are of great interest for energy-storage applications such as for electrode materials in pseudocapacitors. We employ rapid scanning in STEM to both drive and directly observe the atomic scale dynamics behind the transformation of Mn_3_O_4_ into MnO. The results demonstrate we now have the experimental capability to understand the complex atomic mechanisms involved in phase changes and solid state chemical reactions.

Changes in stoichiometry lie at the heart of many materials applications. For instance, batteries commonly rely on the exchange of charged ions between anode and cathode to change their stoichiometries to either store or output energy. Diffraction techniques provide useful information on changes to the average structure, but to resolve the mechanisms through which such changes occur requires local information. The majority of high-resolution *in situ* experiments are performed in conventional TEM instruments with bright-field imaging^1^. Single-shot dynamic TEM^2^ (DTEM) provides phenomenal nanosecond temporal resolution, but changes seen in such phase contrast images often cannot be unambiguously interpreted because of the relatively complicated coherent nature of image formation.

We employ the STEM imaging technique pioneered by Crewe and co-workers^3^. They used the technique to obtain the first TEM images of single atoms and later of the diffusion of individual uranium atoms^4^. STEM imaging has progressed immensely since those early experiments. Aberration correction^5^,^6^,^7^ has not only made atomic resolution imaging routine, but the resulting improved signal to noise ratio allows faster image acquisition enabling the observation of dynamic processes. Furthermore, the incoherent nature of ADF STEM^8^,^9^ makes possible the quantification of images^10^,^11^,^12^,^13^,^14^,^15^ to gain column composition and occupancy information allowing the migrations of atoms between crystallographic sites to be monitored. Spectrum imaging with either electron energy loss spectroscopy (EELS) or energy-dispersive x-ray spectroscopy (EDX) is simultaneously available to provide unambiguous composition and bonding information. These tools make STEM an excellent tool for studying structural and compositional dynamics.

Stroboscopic 4D ultrafast STEM^16^ makes use of laser excitement to achieve up to femtosecond temporal resolution, but is limited to perfectly reversible reactions. In such experiments only one or a few electrons see each reaction and thus millions of cycles are required to form an image. Specially designed heating or cryogenic holders make it possible to control temperature *in situ,* but can compromise the performance of the microscope, reducing stability and maximum resolution. However, there is an alternative source of energy - the electron beam itself. Electron microscopists tend to automatically dismiss data showing beam induced changes in materials, but there is growing recognition that such changes can in fact be informative^17^,^18^,^19^,^20^,^21^,^22^,^23^,^24^. Particularly with the lower accelerating voltages enabled by aberration correction, rather than just knocking atoms directly out of a lattice, the electron beam can provide just enough energy for a system to transition over an energy barrier. In STEM, aberration correction also enables faster rapid scanning, enabling us to capture these transitions in action. Such dynamic ADF STEM imaging has previously been used to capture the motion of impurity atoms and point defects in graphene, boron nitride and carbon nanotubes^12^,^20^,^21^,^25^,^26^,^27^. Beam induced dynamics have also recently revealed the reversible metastable dynamics of a Si_6_ cluster^28^ and the metastable dynamics behind the emission of white light from individual ultrasmall CdSe nanoclusters^29^,^30^. Here we illustrate how the technique can be used to gain insight into the energetics of an irreversible solid state chemical reaction and the resultant phase change.

Figure 1a displays an atomic resolution image capturing the transformation of Mn_3_O_4_ into MnO in progress. The MnO was nucleated by exposing a small region at the edge of the Mn_3_O_4_ to the STEM electron probe for a prolonged period of time. After nucleating the MnO at the edge of the Mn_3_O_4_ flake the MnO spread multiple nanometers towards the centre of the flake as this was the range contained within the field of view during scanning. EELS (figure 1b and Supplementary Information) provides unambiguous identification of these compositions through comparison to reference data^31^. The spinel structure of Mn_3_O_4_ is also easily distinguished from the rock salt structure of MnO, particularly with the sensitivity of ADF imaging to the number of atoms in each atomic column. The image shows that the interface orientation relationship is [110]MnO//[100]Mn_3_O_4_ and (1-10)MnO//(010)Mn_3_O_4_. As indicated in figure 1a, when viewed in this orientation there are three types of Mn columns forming the spinel structure of the Mn_3_O_4_. A and B type columns contain Mn^3+^, while type C columns contain Mn^2+^. Type A columns contain twice as many Mn atoms as B and C type columns and hence appear brighter in the image.

The process of the phase transformation unfolds primarily at the phase front, indicated by the white arrow in figure 1a. By recording a continuous series of rapidly scanned images in this region we provide the energy for the reaction to proceed while simultaneously recording the detailed motions of the atomic columns as the phase front advances. The maximum energy transferred to the Mn atoms is estimated to be approximately 4.4 eV per electron (see supplementary information) with the 100 kV accelerating voltage used here. Generally energies greater than 10 eV are required to displace atoms in bulk crystals^32^, so our beam is gentle enough to stimulate the reaction without damaging the bulk material. A time series of images showing the evolution of the planes adjacent to the phase front is displayed in figure 1c–i. In figure 1c the first plane of Mn columns in the Mn_3_O_4_ adjacent to the phase front are all type B. The next plane down is composed of pairs of type C columns interspersed by type A columns. These two types of planes alternate throughout the rest of the Mn_3_O_4_, and the phase front advance continues by repeating the process exhibited in figure 1c–i. As is particularly clear in the movie (available online) from which these images were extracted, the pairs of C type columns at the phase front appear to dance back and forth between different metastable configurations, partially coalescing and diverging multiple times. Eventually each pair stabilises into a single much brighter column. Meanwhile, the B type columns at the phase front have slowly been increasing in intensity, and continue to do so until they are similarly bright to the A type columns.

We emphasize that due to the incoherent nature of ADF STEM imaging we are able to determine changes in occupation based on the integrated intensities of the columns^33^. For instance the integrated intensity of the type B column pointed to by the red arrow in figure 1c is initially only 63% of the integrated intensity of the type A column immediately below the red arrow. Due to channeling effects one does not expect the A type columns with twice the Mn atoms as the B type columns to have exactly twice the integrated intensity. However, over the time series one sees the B type column increase in intensity until by figure 1i it has reached 97% of the A type column under the red arrow, indicating the formerly B type column has approximately doubled in occupation to match the structure of the A type columns. This is to be expected as MnO only has A type Mn columns. Similarly, the pair of C type columns pointed to by the blue arrow in figure 1c have integrated intensities which are 57% and 65% respectively of those of the A type column below the red arrow. After they merge into a single column the integrated intensity increases to 91% of the A type column under the red arrow and 115% of the A type column immediately to the left of the new Mn column. This indicates that the new merged column is A type, as required for MnO. The variation in intensities observed between columns nominally of the same type is most likely due to the sample increasing in thickness with distance from the edge.

Figure 2 displays a model of the phase front advance based on the dynamic STEM observations. In figure 2a the structure of the first plane of the spinel Mn_3_O_4_ at the phase front only differs significantly from MnO in that its B type Mn columns have half the occupation as the A type column in the MnO. The Mn atoms that are there already occupy positions that should be occupied in MnO. As soon as the vacant sites are filled, the phase front advances. Aside from slight changes in bond lengths, the oxygen atoms do not need to rearrange significantly. This is just what we see with dynamic STEM. The B type columns simply increase in intensity until they reach the intensity of A type columns, indicating that enough additional Mn atoms have diffused into the column to fill the vacant sites. This advances the phase front a single plane. From the ADF imaging we see the C type Mn columns merge into a single column with twice the occupation. Figure 2b–c show how this occurs, advancing the phase front forward another plane. The C type columns contain tetrahedrally coordinated Mn atoms. Each C type column contains Mn atoms spaced midway between those of the adjacent C type column in the [100] direction. All that is needed to convert each pair of C type columns into a single octahedrally coordinated A type column is for them to merge by aligning at their midpoint. As with the previous plane, the oxygen atoms do not need to rearrange significantly. These two basic steps then repeat to continue the advance of the phase front.

DFT calculations of the structures shown in figure 2 confirm the steps in this simple model are energetically favourable. Step one reduces the energy of the system 3.8 eV using the chemical potential for Mn metal to account for the additional Mn atoms (see Supplementary Information). The second step reduces the energy an additional 11.0 eV per unit cell. In reality the material is more than a single unit cell thick, and all the vacant sites at the phase front cannot be filled instantaneously. Dynamic STEM imaging reveals how partial filling results in a slightly more complicated process involving metastable configurations. As the B type columns at the phase front gradually fill, the C type columns in the next plane ahead of the front appear to simultaneously oscillate back and forth as if uncertain of whether to take the MnO or the Mn_3_O_4_ configurations (see movie in supplementary information). This motion is likely due, in part, to the filling in of the vacancies in the B type columns nearer the phase front. Once the B type columns are fully filled the motion of the C type columns also ceases, having fully joined into a single A type column, and the next row of B type columns begins to fill. The reaction rate is therefore likely limited by the diffusion rate of Mn atoms filling the vacant sites in the B type columns.

The source of Mn atoms is likely deoxygenated regions of the Mn_3_O_4_, most likely from the surface. The electron beam is well known to eject oxygen in manganese oxides, and surfaces are typically less stable than the bulk. Mobile Mn atoms can be seen moving along the edge of the material during the dynamic imaging. The edge is the thinnest region in the images and therefore appears darker than the rest of the material, making the mobile atoms difficult to see without over saturating the thicker regions in the images (see supplementary materials for such a movie). A few monolayers of mobile material at the surfaces above and below the thicker regions would not be visible against the contrast of the crystal lattice. However it seems probable that Mn atoms also diffuse along these surfaces, providing the supply of Mn atoms needed to advance the phase front.

In summary, ADF imaging in an aberration corrected STEM has provided a direct view of a solid state chemical reaction in unprecedented detail. By recording a continuous series of rapidly acquired Z-contrast images we simultaneously provide the energy for the transformation of Mn_3_O_4_ into MnO and capture the dynamics of the reaction. By scanning the beam rapidly, the rate of the reaction is kept low enough to resolve the motions of the individual atomic columns as the phase front advances. Furthermore, Z-contrast imaging allows changes in the occupation of individual columns to be quantified. DFT simulations confirm the reaction and the basic steps observed experimentally are energetically favourable. The results illustrate how beam induced changes in materials can be informative, and pave the way towards exploring the complex energy landscapes of other dynamic systems with atomic resolution.

## Methods

The sample was prepared by exfoliation in an ultrasonic bath. The STEM experiments were performed on a Nion UltraSTEM 100 operated at 100 kV equipped with an Enfina EEL spectrometer and an aberration corrector capable of correcting third and fifth order aberrations. Rapid scanning was performed using a dwell time of 2.6 microseconds per pixel and a flyback time of 150 microseconds. For the 512 by 512 images used here, this corresponds to ~0.75 s per frame. Drift correction was performed using the Smart Align software and intensity quantification was performed using the Absolute Integrator software (both available from www.lewysjones.com free of charge for academic/non-commercial use). Density functional theory simulations were performed using the Vienna ab-initio simulation package (VASP) code in the generalized-gradient approximation using the projector-augmented-wave method. Further details are available in the Supplementary Information.

## Author Contributions

T.J.P. initiated the project, performed the STEM experiments and DFT simulations, and wrote the paper, V.N., H.P., J.C., M.C., B.M. prepared the samples, H.P. performed additional electron microscopy characterization and analysis, L.J. performed the column-by-column intensity quantification analysis, V.N. initiated the nanomaterials project at CRANN, and P.D.N. initiated the quantitative STEM project at Oxford and advised on the project and the paper. All the authors read and commented on the manuscript.

## Supplementary Material

Supplementary Information Supplementary Information

Supplementary Information Movie 1: 4-frame running average, 16:9 ratio

Supplementary Information Movie 2: no frame averaging, 16:9 ratio

Supplementary Information Movie 3: 4-frame running average, larger area

## Figures and Tables

**Figure 1:**
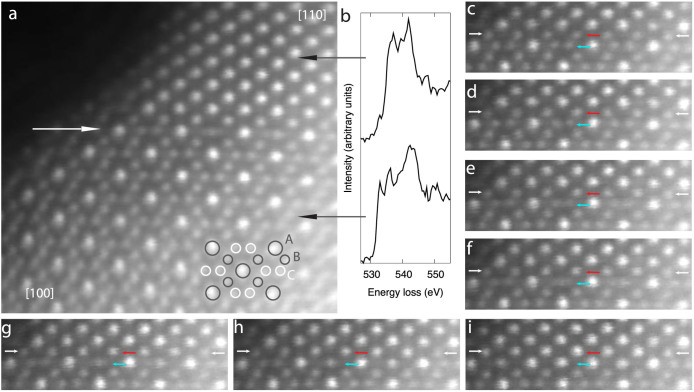
Dynamic ADF STEM imaging of the phase front advance. The phase front is indicated by the white arrow in (a). EELS identifies the new phase seen in the upper part of a as MnO and the original material as Mn_3_O_4_ through the fine structure of the O K-edge (b) and Mn L-edge (Supplementary Information). An overlay in a indicates the configuration of the three types of Mn columns in the spinel structure. A and B type columns contain Mn^3+^, while type C columns contain Mn^2+^. The A-type column contains double the occupancy of the B and C types. (c–i), Time series of ADF images extracted from a movie (available online). The images reveal how the atomic columns rearrange and fill to advance the phase front downwards. In c the small white arrows indicate the position of the first plane of atomic columns in the Mn_3_O_4_ while a small red arrow and a small blue arrow point to a single type B column and a pair of type C columns respectively. The location of the arrows is kept constant throughout the time series for reference. See text for a description of the events observed in the subsequent images.

**Figure 2:**
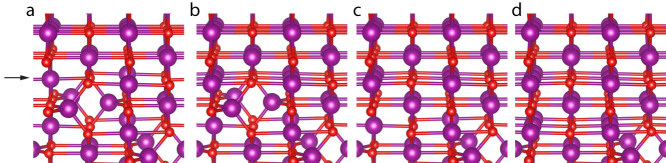
Model of the phase front advance. (a), The first plane of Mn_3_O_4_ is indicated by the black arrow. This plane contains Mn atoms in positions shared by MnO, but the columns contain half as many Mn atoms as in MnO. (b), To advance the phase front forwards, these vacancies must be filled. To convert the next row of columns then requires the pairs of tetrahedral Mn atoms to move into a single column as in (c). (d), Continuing the conversion is then just a repeat of these two steps. Mn and O atoms are shown in purple and red respectively.
