# Supplementary material for: Atomic scale dynamics of a solid state chemical reaction directly determined by annular dark-field electron microscopy
Source: Sci Rep. 2014 Dec 22;4:7555. doi: 10.1038/srep07555 (PMC4273600; doi:10.1038/srep07555)
Supplement: Supplementary Information [file srep07555-s1.doc]

Supplementary information for

Atomic scale dynamics of a solid state chemical reaction directly determined by annular dark-field electron microscopy

Timothy J. Pennycook[[1]](#footnote-2),2*, Lewys Jones^2^, Henrik Pettersson^3,4^, João Coelho^3,5^, Megan Canavan^4^, Beatriz Mendoza-Sanchez^3,5^, Valeria Nicolosi^3,4,5^, and Peter D. Nellist^2,1^

**Sample preparation**

Commercial MnO_2_ powder, purchased from ESPI Metals (Product Number: Kmr1043), was used in order to prepare a 30 ml dispersion of benzyl alcohol with an initial concentration of 10 mg/ml. The dispersion was exfoliated by bath sonication for 3 hrs at a frequency of 80 kHz and power of 40% (Fisherbrand FB11207 Sonic Bath). After sonication, the dispersion was then centrifuged at 3000 rpm for 60 mins (Heraeus Multifuge X1 Centrifuge). Small amount (10 – 12 drops) of the dispersion was pipetted using glass pipette onto a holey carbon film on 400 mesh copper grid, which was left to be baked in order to remove residual solvent.

**TEM investigation**

The flakes obtained after exfoliation generally had lateral sizes of 5 – 20 nm, approximately, and occasionally larger flaks in the range of 50 – 100 nm. The lager flakes were confirmed, by the conventional TEM (FEI Titan 80 – 300 FEG TEM) and EDX, to be MnO_2_ (mostly (110) oriented), while the smaller flakes consisted of polycrystalline MnO_2_ where substantial amounts of spinel Mn_3_O_4_ and MnO were found to be present as secondary phases, especially along the flake rims.

**Aberration corrected STEM Imaging**

The flakes were imaged in a Nion UltraSTEM 100 operated at 100 kV and fitted with an ultrahigh vacuum system, a cold field emission electron source, a corrector of third and fifth order aberrations, and equipped with an Enfina EEL spectrometer^1^. The probe convergence angle was approximately 30 mrad. Although the precise extraction voltage and beam current were not recorded, the probe current under these conditions is typically around 80 pA. The EELS collection angle was calibrated to 36 mrad. The high angle annular dark field detector collection range was approximately 80-240 mrad. The images shown in Fig. 1 of the main text were extracted from a time series of 512 by 512 pixel HAADF images using a short 2.6 microseconds per pixel dwell time and a flyback time of 150 microseconds. This equates to ~0.75 s per frame, allowing us to capture approximately 19 frames per plane the phase front advances. In order to improve the signal to noise ratio, the images shown in figure 1 of the main text are the result of averaging over four sequential frames after correcting for drift with the in-house created Smart Align software (available from [www.lewysjones.com](http://www.lewysjones.com/) free of charge for academic / non-commercial use). The images in figure 1 c-i have also been cropped from the full 512 by 512 pixel images to focus on the dynamics occurring at the phase front. Movies created from the drift corrected time series of images are available online.

**Aberration corrected STEM Quantification**

The frames shown in figure 1 c-i were analysed quantitatively. To achieve this a ‘vacuum level’ was first subtracted from the images determined using the region shown in the upper left of figure 1 a. This subtraction yields data whose values are proportional to the sample scattering. Next an automated peak-finding algorithm was used to identify the positions of the atomic columns, as well as to define Voroinoi cells that encompass the scattering attributed to each column. The intensity within each cell was then integrated to yield the measured scattering from each column. These peak-finding, cell creation and integration steps were all performed using the in-house Absolute Integrator software (available from [www.lewysjones.com](http://www.lewysjones.com/) free of charge for academic / non-commercial use).

**Estimation of the maximum energy transferred to the Mn atoms from the electron beam**

The maximum energy transfer occurs when an electron in the electron beam directly impacts an atom. The exact relativistic kinematics gives^2,3^

where M is the nuclear mass, E_0_ is the energy of the incident electron, m_0_ is the electron mass. As m_0_ << M and E_0_ << Mc^2^ this can be simplified to

which for Mn atoms and the 100 keV accelerating voltage used for the STEM imaging is 4.385 eV.

**Density functional theory simulations**

The simulations were performed using density functional theory^4,5^ in the generalized-gradient approximation and the projector-augmented-wave method^6^ with a plane-wave basis as implemented in the Vienna ab initio simulation package (VASP) code^7^. A supercell was constructed in a slab configuration using the lateral lattice parameters of the Mn_3_O_4_ unit cell. One and a half unit cells of Mn_3_O_4_ were interfaced with a one unit cell thick layer of MnO in the interface orientation relationship [110]MnO//[100]Mn_3_O_4_ and (1-10)MnO//(010)Mn_3_O_4_. A 13.5 Å long volume of vacuum was used to separate the surfaces of the slab. A second structure was created by modifying the initial supercell by adding in two Mn atoms at the interface as shown in figure 2b of the main text. A third structure was created by merging the C-type columns at the interface in the second structure, as shown in figure 2c of the main text. The total energy of these structures was then calculated using spin polarization, up to a 400 eV energy cutoff and with up to a 6x6x1 Monkhorst-Pack k-point mesh (6x6 laterally, with one division for the much longer third dimension of the supercell) centered at the Γ point. In order to account for additional Mn atoms the difference in energy between structures one and two was calculated as

where *E_1_* and E_2_ are the total energies of the two structures, n is the number of additional Mn atoms in the second structure per supercell (two), and μ_Mn_ is the chemical potential for Mn, the total energy per atom in the Mn reservoir, which we take to be the total energy per atom of Mn metal. The total energy per Mn atom in body centred cubic Mn metal was calculated with up to a 37x37x37 Monkhorst-Pack k-point mesh and an energy cutoff of up to 270 eV.

References:

1. Krivanek, O. L. *et al.* An electron microscope for the aberration-corrected era. *Ultramicroscopy* **108,** 179–195 (2008).

2. Banhart, F. Irradiation effects in carbon nanostructures. *Reports on Progress in Physics* **62,** 1181–1221 (1999).

3. Egerton, R. F., McLeod, R., Wang, F. & Malac, M. Basic questions related to electron-induced sputtering in the TEM. *Ultramicroscopy* **110,** 991–997 (2010).

4. Hohenberg, P. & Kohn, W. Inhomogeneous electron gas. *Phys. Rev* **136,** 864–871 (1964).

5. Kohn, W. & Sham, L. Self-consistent equations including exchange and correlation effects. *Phys. Rev* **140,** A1133–A1138 (1965).

6. Blochl, P. Projector Augmented-Wave Method. *Phys Rev B* **50,** 17953–17979 (1994).

7. Kresse, G. & Furthmuller, J. Efficient iterative schemes for ab initio total-energy calculations using a plane-wave basis set. *Phys Rev B* **54,** 11169–11186 (1996).

1. ^1^ SuperSTEM Laboratory, STFC Daresbury, Keckwick Lane, Warrington WA4 4AD, United Kingdom.^2^ Department of Materials, University of Oxford, Parks Road, Oxford OX1 3PH, United Kingdom. ^3^ Centre for Research on Adaptive Nanostructures and Nanodevices (CRANN), Trinity College Dublin, Dublin 2, Ireland. ^4^ School of Physics, Trinity College Dublin, Dublin 2, Ireland. ^5^ School of Chemistry, Trinity College Dublin, Dublin 2, Ireland. [↑](#footnote-ref-2)
